# Association of miR-146a, miR-149, miR-196a2, miR-499 gene polymorphisms with ischemic stroke in a Chinese people

**DOI:** 10.18632/oncotarget.18333

**Published:** 2017-06-01

**Authors:** Hong-Cheng Luo, Qi-sheng Luo, Chun-Fang Wang, Ming Lei, Bei-Lin Li, Ye-Sheng Wei

**Affiliations:** ^1^ Department of Laboratory Medicine, Affiliated Hospital of Youjiang Medical University for Nationalities, Baise, 533000, Guangxi, China; ^2^ Department of Neurosurgery, Affiliated Hospital of Youjiang Medical University for Nationalities, Baise, 533000, Guangxi, China

**Keywords:** allele, genotype, ischemic stroke, microRNA, single nucleotide polymorphism

## Abstract

This study aimed to investigate genetic polymorphisms of *miR-146a*, *miR-149*, *miR-196a2*, and *miR-499* and genetic susceptibility of ischemic stroke in the population of Guangxi in China. A case–control study was used to investigate miRNAs genetic polymorphisms in 298 patients with ischemic stroke and 303 healthy controls. Single-base extension polymerase chain reaction genotyping principle was used to detect genetic polymorphisms of miRNAs,and the relationship of genotype in each group and blood lipid was compared and analyzed. The genetic polymorphism of *miR-499A>G* (rs3746444) was associated with ischemic stroke (P < 0.05), and the risk of ischemic stroke was high in patients with G allele (OR = 1.455; 95% CI = 0.531–2.381; P = 0.039) and AG (OR = 1.339; 95% CI = 1.126–1.967; P = 0.037) genotype. The levels of low-density lipoprotein cholesterol, very-low-density lipoprotein cholesterol, homocysteine, and lipoprotein in the ischemic stroke group were higher than those in the control group (P < 0.05). The genetic polymorphism of *miR-499A>G* (rs3746444) was related to ischemic stroke, and G allele and AG genotype may increase the risk of ischemic stroke in the population of Guangxi in China.

## INTRODUCTION

Ischemic stroke (IS) is a cardiovascular disease that seriously threatens the health of middle-aged and elderly people. In China, due to its high morbidity, high disability rate, high mortality, and high recurrence rate, IS has surpassed the morbidity of heart disease and tumors [1], which brings tremendous economic pressure and health burden to the family and the society [2–4]. Multiple factors including smoking, hypertension, diabetes, hyperlipidemia, and high homocysteine content are risk factors for IS [5]. However, these risk factors can only explain a small part of the etiology. IS is a complex disease induced by the combined action of a variety of genetic and environmental factors, and genetic mutations play a crucial role in its development [6–8]. Nevertheless, the exact molecular mechanism underlying the development of IS has not yet been determined.

MicroRNAs (miRNAs) are endogenous noncoding small-molecule RNAs mainly composed of 19–25 nucleotides in eucaryon. Through complementary binding with 3′ untranslated regions in the target gene of mRNA, miRNAs can post-transcriptionally regulate the gene expression. Several studies have shown that miRNAs are involved in a series of important life processes, including cell proliferation, development, differentiation, apoptosis, and metabolism [9, 10]. Genetic mutations existing in miRNA sequences affect the expression of mature miRNAs, thereby influencing the expression of target genes. Moreover, studies have shown that miRNAs are closely associated with growth/development, differentiation, maturation, and lesion of the nervous systems [11].

Single nucleotide polymorphism (SNP) is one of the most common types of genetic variation. SNP that exists in the precursor miRNAs (pre-miRNAs) can affect the mature expression of miRNAs, and then change multiple biological functions mediated by the target genes. Currently, many studies have shown that SNP loci that exist in the four precursor miRNAs, including *miR-146aC>G* (rs2910164), *miR-149T>C* (rs2292832),*miR-196a2T>C*(rs11614913), and *miR-499A>G* (rs3746444), are closely associated with various diseases that are seriously harmful to human health, such as tumors, hypertension, cardiovascular disease, and diabetes [12–15]. Recently,the *miR-146aC>G*, *miR-149T>C*, *miR-196a2T>C*, and *miR-499A>G* alleles are possible genetic predisposing factors [16–20]. These 4 miRNAs can affect vascular damage responses [21–24]. Moreover, The *miR-146a, miR-149, miR-196a2, and miR-499* alleles are closely associated with regulation of tumor necrosis factor-α(TNF-α) [21], methylenetetrahydrofolate reductasefour [22], and C-reactive protein (CRP) [24], These miRNA targets are related to thrombosis or inflammation pathways in the circulation system. So far, the correlation of genetic polymorphisms of *miR-146aC>G, miR-149T>C, miR-196a2T>C, and miR-499A>G* in the population Guangxi in China has not been reported both at domestic and foreign. Therefore, this study aimed to investigate the correlation of IS and SNP of the four miRNAs in the population of Guangxi.

## MATERIALS AND METHODS

### Subjects of Study

The study population comprised 298 patients with IS and 303 controls. All the population were recruited from the department of neurology, Affiliated Hospital of Youjiang Medical University for Nationalities, Guangxi, China between January 2015 and June 2015. All of them had the first-time IS. The study was approved by the Ethics Committee of You Jiang Medical College and conformed to the guidelines set forth by the Declaration of Helsinki. The diagnosis met the criteria approved at the Fourth National Cerebrovascular Disease Conference in 1995. The patients were diagnosed by at least two experienced clinical neurologists through brain computed tomography (CT) and (or) magnetic resonance imaging (MRI). Patients with IS caused by cardiovascular malformations or tumors, trauma, blood diseases, drugs, and infectious diseases were excluded. The exclusion criteria of the population in the control group were the same as that in the IS group. The patients had no history of stroke or severe genetic diseases in the family.

### Research methods

Extraction of genomic DNA: EDTA-K2-mediated anti-coagulated venous blood (3mL) was obtained from people who received physical examination. A whole-blood DNA extraction kit (Shenzhen Yaneng Bioscience Co., Ltd., China) was used to extract the genomic DNA of whole blood according to the manufacturer instruction. The extracted DNA was stored at −70°C.

The design and synthesis of the primers: According to the GeneBank of National Center for Biotechnology Information, the human full-length sequences of the four SNP loci [*miR-146aC>G* (rs2910164), *miR-149T>C*(rs2292832), *miR-196a2T>C*(rs11614913), and *miR-499 A>G* (rs3746444)] were used. Primer3.0 was used to design the primers, and the primers were synthesized by Sangon Biotech (Shanghai) Co., Ltd. Sequences of each primer are shown in Table 1.

**Table 1:** Primers sequences of each SNP locus in miR-146a, miR-149, miR-196a2, and miR-499

SNP loci	Sequences of PCR primers		Sequences of extension primers
rs2292832	Upstream primer	5′-TCTGGCTCCGTGTCTTCACTCC-3′	5′-TTTTTTTTCGGCGACCTGCGTTGTTCC-3′
	Downstream primer	5′-GGCCCGAAACACCCGTAAGATA-3′	
rs3746444	Upstream primer	5′-CTGGGAGACAGACCCTCCCTCT-3	5′-TTTTTTTTTTTTTTTTTTTTTTTTTGTTTAACTCCTCTCCACGTGAAC-3′
	Downstream primer	5′-GCCCTGCACTTTTGCTCTTTCA-3	
rs2910164	Upstream primer	5′-AGGAAGCAGCTGCATTGGATTT-3′	5′-TTTTTTTTTTTTTTTTTTTTTTTTTTTTTTTTTTTTTTTGGGTTGTGTCAGTGTCAGACCT-3′
	Downstream primer	5′-GTCCTCAAGCCCACGATGACAG-3′	
rs11614913	Upstream primer	5′-CCCCTTCCCTTCTCCTCCAGAT-3′	5′-TTTTTTTTTTTTTTTTTTTTTTTTTGAACTCGGCAACAAGAAACTG-3′
	Downstream primer	5′-TTGTTCTGCAACCCCACTCACA-3′	

The amplification of polymerase chain reaction (PCR): Four SNP loci, including miR-146aC>G (rs2910164), *miR-149T>C* (rs2292832), miR-196a2T>C (rs11614913), and *miR-499A>G* (rs3746444) were used. Reaction system for each PCR amplification contained a total volume of 20 mL, including 2 mL of PCR buffer (1× GC buffer I, Takara), 2.0 mL of 2.5 mmol/L Mg^2+^, 2.0 mL of 2 mmol/L dNTP, 1 μL of 0.2 mol/L upstream primer, 1 μL of 0.2 mol/L downstream primer, 1.0 U of polymerase, and 1.0 μL of template DNA. If the volume was less than 20 μL, then sterile double-distilled water was used to complement. PCR product was obtained using Multiplex PCR Master Mix with HotStar Taq DNA Polymerase (Qiagen). Then, 8 μL of PCR product was purified with 0.5 U of shrimp alkaline phosphatase (SAP) enzyme (Promega) and 4 U of exonuclease I (Epicentre), and incubated at 37°C for 60 min using the technique of SNaPshot Multiplex Kit (ABI), followed by incubation at 75°C for 15 min for extension reaction. Finally, the extension product was sequenced by electrophoresis using ABI3730XL sequencer after purification with SAP. GeneMapper4.0 (ABS) was used to analyze SNP genotype. The reaction condition of PCR was as follows: 95°C for 2 min; 11-cycle reaction (94°C for 20 s, 65°C for 40 s, 72°C for 1 min); 24-cycle reaction (94°C for 20 s, 59°C for 40 s, 72°C for 1 min); 72°C for 2 min; 4°C.

### The measurement of serum homocysteine (Hcy) and blood lipid level

Heparin-anticoagulated blood was collected from patients within 24 h after being hospitalized, and then the blood was centrifuged at 3000 rpm for 5 min to separate the serum to be used in the measurement of blood lipid and Hcy. Enzymatic measurement was used for detecting TC, TG, and Hcy, and the method of masking was used to directly measure high-density lipoprotein cholesterol (HDL-C), low-density lipoprotein cholesterol (LDL-C), very-low-density lipoprotein cholesterol (VLDL-C), and lipoprotein a [Lp(a)] (Hitachi 7600 automatic biochemical analyzer).

### The relative expression levels of miR-499 and C-reactive protein(CRP) in patients with IS harboring different alleles

Total RNA was extracted from 1ml of 100 plasma samples(50 AA and 50 AG+GG genetype) using miRCURY Exosome Isolation Kit (Takara, Dalian, China) according to the manufacturer's instructions .5.0μg of total RNA was reverse transcribed into cDNA using poly(A) polymerase and Mir-X miRNA First-Strand Synthesis Kit(Takara, Cat. No. 638313).Real-time reverse transcription PCR(RT-PCR) and Mir-X miRNA qRT-PCR SYBR Kit (Takara, Cat. No. 638314) were performed to validate expression levels of *miR-499*.Quantitative PCR was performed on ABI 7900HT real-time PCR machine (Applied Biosystems, CA, USA), three shrimps were analyzed for each genetype of plasma samples. U6 RNA was used as an endogenous control,the primers were purchased from Takara., China. The relative serum levels of *miR-499* expression were calculated using the delta-delta Ct method (2−ΔΔCt). The levels of serum CRP in 215 AA genotypes were compared with 83 (AG + GG) genotype by immunoturbidimetry Method(Hitachi 7600 series).

### Statistical analysis

SPSS13.0 (SPSS, Inc., IL, USA) was used to conduct statistical analysis. Allele and genotype frequencies of the IS and control groups used the χ^2^ or Fisher's exact test. Measurement data were represented by x ± s, and categorical data were represented by χ^2^. OR value and 95% confidence intervals were used to evaluate the correlation of each allele and genotype distribution frequencies. Multivariate logistic regression was used to evaluate the relationship between the risk factors (gender, age, smoking situation, diabetes, hypertension, and Hcy) and the disease. χ^2^ was used to evaluate whether the allele and genotype in the control group met the conditions of Hardy–Weinberg equilibrium. All the tests were bilateral detections, and *P* < 0.05 was considered to be statistically significant.

## RESULTS

### Clinical data of the enrolled population

Comparison of clinical indicators between the IS and control groups is shown in Table 2. Patients with IS were predominantly smokers. So, patients with hypertension or diabetes were significantly more than those in the control group (*P* < 0.05). The levels of LDL-C, VLDL-C, Hcy, and Lp(a) were significantly higher than those in the control group (*P* < 0.05), but the level of HDL-C in patients with IS was significantly lower than that in the control group (*P* < 0.05).

**Table 2:** Comparison of basic clinical data between the IS and control groups

Groups	IS group (298 cases)	Control group (303 case)	χ^2^	*P*
M/F (cases)	196/102	181/122	2.341	0.126
Age (year)	60.70 ± 12.33	60.17 ± 10.32	1.030	0.303
Smoking (cases)	96 (32.2)	55 (18.2)	15.80	< 0.001
Hypertension (cases)	204 (68.5)	75 (24.8)	115.38	< 0.001
Diabetes (cases)	79 (26.5)	26 (8.6)	33.50	< 0.001
TC (mmol/L)	5.10 ± 1.18	5.51 ± 0.32	0.087	0.930
TG (mmol/L)	1.85 ± 1.65	2.41 ± 0.10	0.226	0.821
HDL-C (mmol/L)	1.23 ± 0.31	1.73 ± 0.47	15.498	< 0.001
LDL-C (mmol/L)	2.30 ± 1.91	2.36 ± 0.99	5.078	< 0.001
VLDL-C (mmol/L)	0.86 ± 0.77	0.69 ± 0.51	3.219	0.001
HCY (μmmol/L)	13.98 ± 7.15	8.96 ± 7.02	5.084	< 0.001
LP(a) (mg/dL)	24.30 ± 20.35	12.43 ± 11.51	8.099	< 0.001

### The genotyping result of *miR-146aC>G*, *miR-149T>C*, *miR-196a2T>C*, and *miR-499A>G*

**Figure 1:**
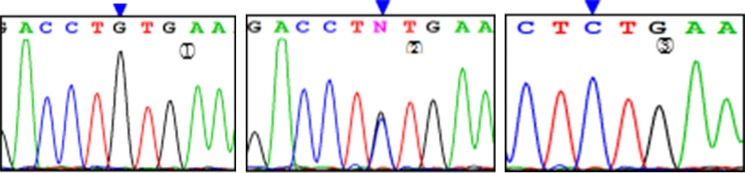
Sequencing diagram of miR-146C>G (rs2910164) Note:①, ②, and ③ represent genotypes of GG, CG, and CC, respectively; arrows are loci of genetic mutations.

**Figure 2:**
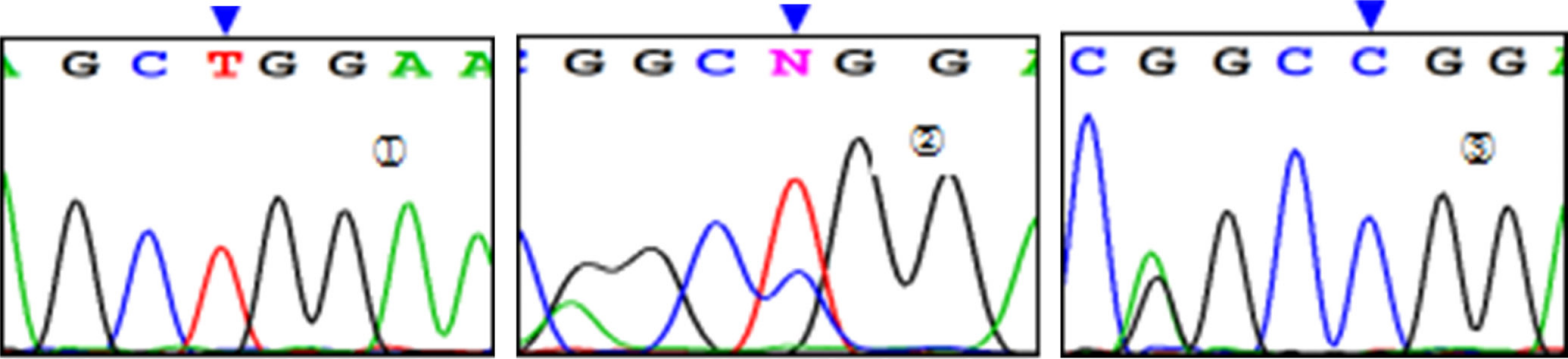
Sequencing diagram of miR-149T>C (rs2292832) Note: ①, ②, and ③ represent genotypes of TT, CT, and CC, respectively; arrows are loci of genetic mutations.

**Figure 3:**
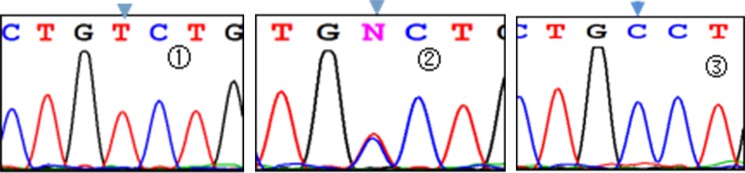
Sequencing diagram of miR-196a2T>C (rs11614913) Note: ①, ②, and ③ represent genotypes of TT, CT, and CC, respectively; arrows are loci of genetic mutations.

**Figure 4:**
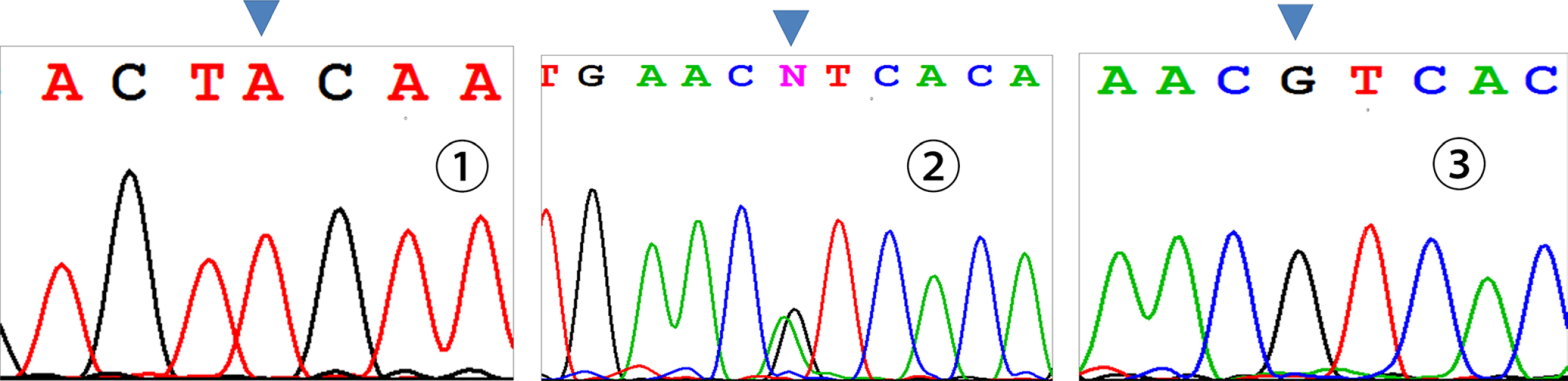
Sequencing diagram of miR-499A>G (rs3746444) Note: ①, ②, and ③ represent genotypes of AA, AG, and GG, respectively; arrows are loci of genetic mutations.

Three genotypes were detected by SNP of *miR-146aC>G* gene, including GG, GC, and CC (Figure 1); *miR-149T>C* gene also had three genotypes, including CC, CT, and TT (Figure 2). Moreover, CC, CT, and TT (Figure 3) were the genotypes for *miR-196a2T>C*, while AA, AG, and GG (Figure 4) were the genotypes for *miR-499A>G*.

### Comparison of the allele and genotype distribution frequencies between the IS and control groups

Table 4 shows the allele and genotype distribution of *miR-146aC>G*, *miR-149T>C, miR-196a2T>C, and miR-499A>G* in the IS and control groups. Genotype distribution of the control group met the requirements of the Hardy–Weinberg equilibrium (rs2910164C>G, *P* = 0.672; rs2292832T>C, *P* = 0.447; rs11614913T>C, *P* = 0.385; rs3746444A>G, *P* = 0.131). After multivariate logistic regression analysis (including gender, age, smoking, hypertension, diabetes, high Hcy, and high lipoprotein), statistical differences were found in the allele and genotype distribution of *miR-499A>G* in the IS group [comparison of *miR-499A> G* allele and genotype between the IS and control groups showed significant differences [AG vs AA, adjusted OR value (AOR) = 1.339, 95% CI = 1.126– 1.967, *P* = 0.037; G vs A, AOR = 1.455, 95% CI = 1.019–2.381, *P* = 0.037)] compared with the control group. No statistical difference in the genetic polymorphisms of *miR-146aC>G, miR-149T>C,* and *miR-196a2T>C* was found between the IS and control groups (*P* > 0.05).

**Table 3:** Comparison of the levels of blood lipid, Lp(a), and Hcy of each genotype in the IS group $\left(\bar{x}\pm s\right)$

Items	rs2292832 T/C			rs3746444 A/G			rs2910164 C/G			rs11614913 T/C		
	TT	CT + CC	P value	AA	AG + GG	P value	CC	CG + GG	P value	TT	CT + CC	P value
	131 cases	167 cases		215 cases	83 cases		129 cases	169 cases		73 cases	225 cases	
TC (mmol/L)	5.17 ± 1.29	5.05 ± 1.11	0.38	5.07 ± 1.21	5.21 ± 1.04	0.44	5.16 ± 1.25	5.05 ± 1.14	0.46	5.00 ± 1.14	5.13 ± 1.20	0.35
TG (mmol/L)	1.90 ± 1.50	1.81 ± 1.74	0.62	1.87 ± 1.04	1.75 ± 1.18	0.63	1.95 ± 1.40	1.78 ± 1.02	0.91	1.72 ± 1.04	1.88 ± 1.81	0.46
HDL-C (mmol/L)	1.22 ± 0.26	1.23 ± 0.33	0.71	1.22 ± 0.30	1.25 ± 0.31	0.51	1.21 ± 0.32	1.24 ± 0.30	0.50	1.25 ± 0.32	1.22 ± 0.30	0.42
LDL-C (mmol/L)	2.94 ± 0.84	3.03 ± 2.36	0.70	2.98 ± 2.08	3.03 ± 0.81	0.87	2.95 ± 0.88	3.02 ± 2.35	0.72	2.81 ± 0.86	3.05 ± 2.14	0.33
VLDL(mmol/L)	0.86 ± 0.68	0.85 ± 0.83	0.93	0.80 ± 0.54	0.80 ± 0.54	0.51	0.91 ± 0.61	0.83 ± 0.50	0.24	0.80 ± 0.49	0.88 ± 0.84	0.40
HC(μmmol/L)	14.27 ± 7.44	13.78 ± 6.98	0.56	14.22 ± 7.57	14.22 ± 7.57	0.78	13.90 ± 6.77	14.02 ± 7.40	0.88	14.94 ± 7.88	13.66 ± 6.88	0.23
LP(a)(mg/dL)	22.90 ± 18.51	25.20 ± 21.45	0.34	23.82 ± 19.52	26.47 ± 23.81	0.39	23.38 ± 18.66	24.89 ± 21.40	0.48	24.65 ± 22.00	24.18 ± 20.23	0.96

**Table 4:** Comparison of genotype distribution frequencies of the allele and genotypes of polymorphic loci in the IS and control groups

SNPs	Stroke (298 cases)	Control (303 cases)	AOR (95% CI)	*P* value
miR-149T>C				
TT	131 (44.0)	121 (39.9)	1.000 (reference)	
CT	127 (42.6)	136 (44.9)	1.108 (0.879–1.647)	0.756
CC	40 (13.4)	46 (15.2)	1.226(0.963–1.726)	0.301
Dominant model (TT vs CT + CC)			0.958(0.713–1.295)	0.363
Recessive model(TT + CT vs CC)			0.776(0.659–1.375)	0.575
T	389 (65.3)	378 (62.4)	1.000 (reference)	
C	207 (34.7)	228 (37.6)	1.156 (0.916–1.597)	0.325
*HWE P*	0.300	0.447		
miR-499A>G				
AA	215 (72.1)	244 (80.5)	1.000 (reference)	
AG	78 (26.2)	53 (17.5)	1.339 (1.126–1.967)	0.037
GG	5 (1.7)	6 (2.0)	1.077 (0.361–2.024)	0.669
Dominant model (AAvs AG + GG)			1.621 (1.079–2.516)	0.027
Recessive model (AA+ AG vs GG)			0.879 (0.531–2.557)	0.665
A	508 (85.2)	541 (89.3)	1.000 (reference)	
G	88 (14.8)	65 (10.7)	1.455 (1.019–2.381)	0.039
*HWE P*	0.491	0.131		
miR-146aC>G				
CC	129 (43.3)	119 (39.3)	1.000 (reference)	
CG	130 (43.6)	139 (45.9)	1.163(0.939–1.681)	0.596
GG	39 (13.1)	45 (14.9)	0.991(0.533–1.475)	0.557
Dominant model (CC vs CG + GG)			1.015 (0.623–1.273)	0.329
Recessive model (CC + CG vs GG)			1.006(0.543–1.319)	0.317
C	388 (65.1)	377 (62.2)	1.000(reference)	
G	208 (34.9)	229 (37.8)	1.091 (0.075–1.451)	0.776
*HWE P*	0.490	0.672		
miR-196a2T>C				
TT	73 (24.5)	75 (24.8)	1.000 (reference)	
CT	138 (46.3)	159 (52.5)	1.257 (0.976–2.058)	0.190
CC	87 (29.2)	69 (22.8)	1.339 (0.894–1.996)	0.216
Dominant model (TTvsCT + CC)			0.803 (0.576–1.127)	0.249
Recessive model (CCvsCT +TT)			1.376 (0.759–1.963)	0.173
T	284 (47.7)	309 (51.0)	1.000 (reference)	
C	312 (52.3)	297 (49.0)	1.035 (0.799–1.541)	0.559
*HWE* P	0.215	0.385		

*Note*: HWE P: Wendy–Harberger's law; miRNA: microRNA; 95% CI: 95% confidence interval; AOR: adjusted OR value.

Corrected factors of the AOR include gender, age, smoking, hypertension, diabetes, high Hcy, and high Lp(a).

### Comparison of blood lipid, Lp(a), and Hcy in each genotype locus in the IS group

No statistical difference in blood lipid, lipoprotein (a), and Hcy was observed in each genotype of *miR-146aC>G, miR-149T>C, miR-196a2T>C*, and *miR-499A>G* in the IS group (*P* > 0.05, Table 3 *Comparison of relative expression levels of miR-499 and C-reactive protein(CRP) in patients with IS harboring different alleles*

**Figure 5:**
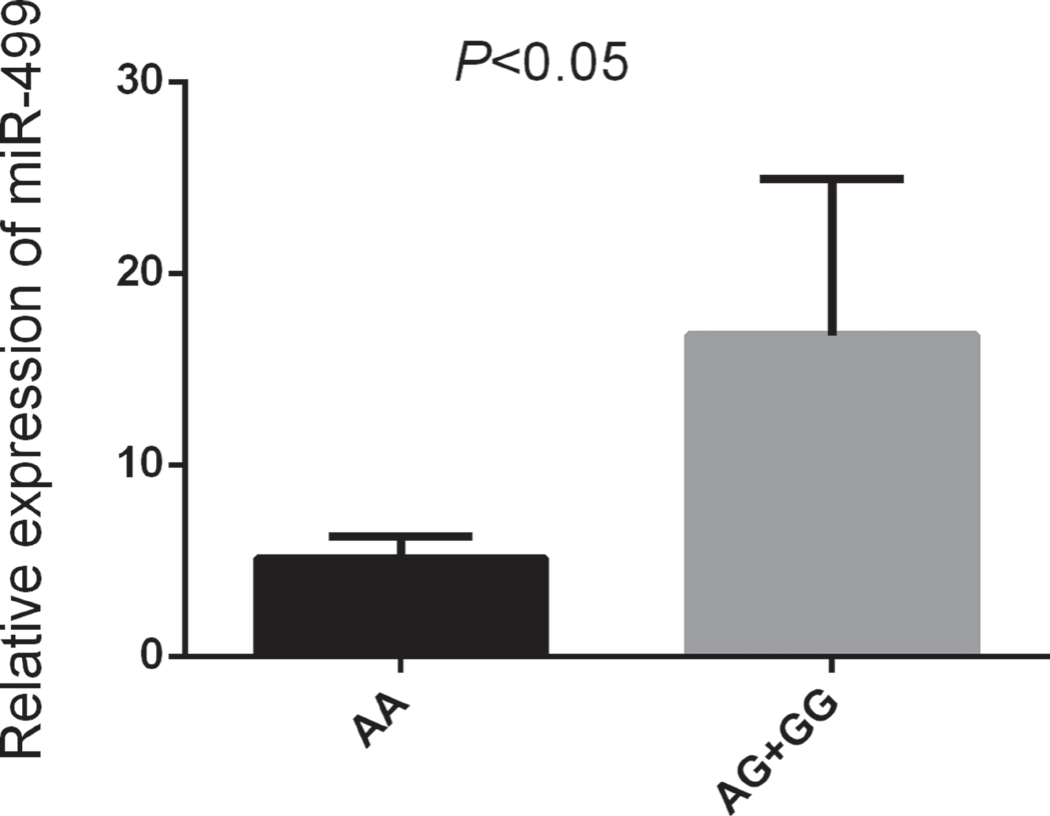
Relative expression of miR-499 Note:Increased level of miR-499 in IS patients carrying the AG/GG (*n* = 50) compared with AA (*n* = 50) (*P* < 0.05).

**Figure 6:**
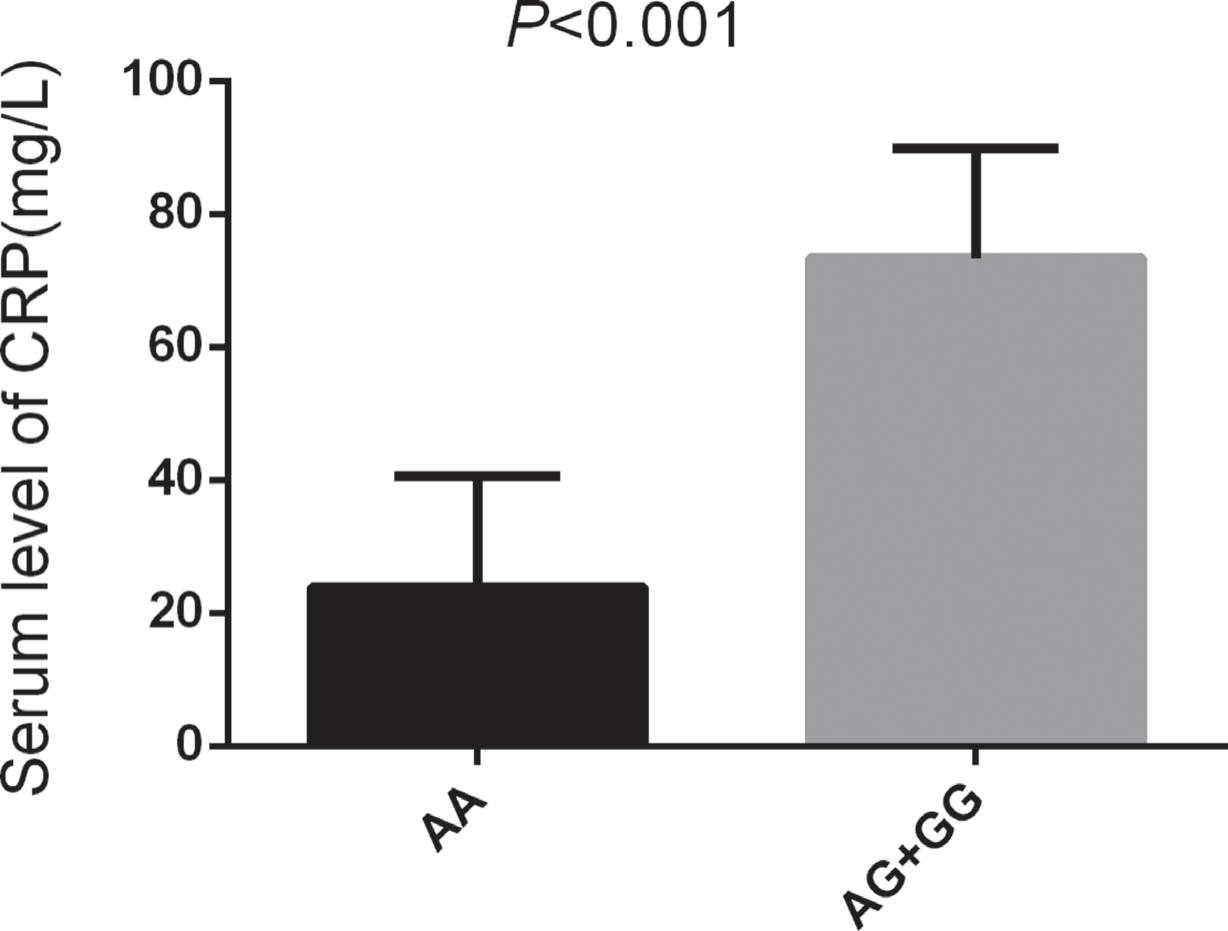
Serum level of CRP in different alleles of miR-499 Note: Significant difference of serum CRP level between AA (*n* = 215) and AG/GG (*n* = 83) in IS patients (*P* < 0.001).

The results show that patients carrying the rs3746444AG/GG genetype had a higer level of miR-499 and CRP compared with those carrying the rs3746444AA genetype (*P* < 0.05) (Figures 5, 6).

## DISCUSSION

As the major type of stroke, IS refers to cerebral necrosis caused by stenosis or occlusion of the cerebral artery, and cerebral insufficiency, which is a complex neurological disease induced by interaction of genes with the environment that causes permanently disabling disease [6, 25–26]. One in six people in the world suffer from stroke [27], and IS seriously increases the burden on economy and medical resource in the world [28]. However, traditional diagnosis relies on CT or MRI, which cannot completely diagnose all types of strokes. Satisfactory clinical results cannot be obtained especially regarding the risk factors for IS. The role of genetic factors is increasingly evident in the state of the disease, especially in stroke [29]. MiRNAs are small-molecule RNAs recently found to be involved in the physiological and pathological processes of various diseases, including tumors [30], functions of immune system [31], cell proliferation [9], cardiovascular diseases [13], and nervous system diseases [32]. MiRNAs have a high degree of stability, tissue specificity, specificity of cell expression, and easy detectability in the human circulation. Therefore, miRNAs have become a molecular marker for the clinical diagnosis in many diseases, especially for early damage of the nervous system, cardiovascular diseases, and early painless diagnosis of IS [25, 33].

MiRNAs can regulate about 30% human gene expression [34]. SNP existing in pre-miRNAs or genetic mutations can affect the expression of mature miRNAs or cause mutative expression [35]. SNP existing in miRNAs are closely related to traditional risk factors for IS, such as atherosclerosis, hypertension, hyperlipidemia (high cholesterol), and diabetes [36–39]. Based on preliminary studies, four miRNAs were selected [*miR-146aC>G* (rs2910164), m*iR-149T>C* (rs2292832), *miR-196a2T>C* (rs11614913), and *miR-499A>G* (rs3746444] to investigate the genetic susceptibility of SNP and IS in the population of Southwest region of Guangxi in China. It was found that *miR-499A>G* (rs3746444) was correlated with IS in the population Southwest region of Guangxi in China (*P* < 0.05). The risk of stroke would increase in people with G allele (OR = 1.455; 95% CI = 0.531–2.381) and AG genotype (OR = 1.339; 95% CI = 1.126–1.967; *P* = 0.037). We found that carrying the rs3746444AG/GG genetype had a higer level expression of *miR-499*, These findings were similar to the findings of the study by Liu et al. [40] that genetic polymorphism of *miR-499A>G* (rs3746444) might increase the risk of stroke in the Sichuan Han population. The rs3746444 A/G polymorphism association with ischemic stroke risk might be involved in thrombosis and/or inflammation pathways in the circulation system. The changes in *pre-miR-499*A: U or G: U base pairing would affect the expression of the target genes of mature miR-499 and biological functions [41, 42], cell hypoxia, or apoptosis caused by overexpression of miR-499 [43], and all these can increase the risk of stroke. The study found that carrying the rs3746444 AA had a lower level of serum CRP compared to those rs3746444AG/GG, miR-499 would increase the concentration of serum or plasma C-reactive protein, causing increased risk factors for stroke, such as inflammation, hypertension, and hyperlipoidemia. In summary, the pathological process of *miR-499* in stroke and its potential mechanism are still unclear, but the mechanism that can increase the incidence of stroke through regulating the level of traditional risk factors for IS has been recognized. It is of immense significance in searching new diagnostic markers of stroke through in-depth study of the relationship between genome-wide associative analysis and stroke.

This study showed no correlation between IS and genetic polymorphisms of *miR-146a, miR-149*, and *miR-196a2* in the population of Guangxi in China. However, the study by Jeon et al. [44] suggested that *miR-146a* G allele was associated with the morbidity of IS in the Korean population. A study by Zhu et al. [45]demonstrated that *miR-146a* C allele and CC genotype could increase the risk of stroke in the Han population in northern China. Qu et al. [46]reported that *miR-146a* (rs2910164) can be used as a diagnostic marker for the prognosis of stroke in the Asian population. Huang et al. [47] claimed that *miR-146a* (rs2910164) was significantly correlated with the stroke in the Han population in Shenzhen, China. Moreover, Chen et al. [48] indicated that *miR-149* was associated with the risk of stroke in the Han population in Jiangsu, China. However, no study has elucidated that the genetic polymorphism of *miR-196a2* is associated with stroke. The results of the aforementioned studies varied in different ethnic groups in different countries, which might be due to regional differences and different lifestyles, environment, stresses. And it suggested that the polymorphisms might have tissue-specific and different effect on human diseases in different organs, reflecting the diversities of the etiological factors for different diseases.

Ischemic stroke is a complex disease caused by a variety of factors. It can be caused by the interaction among multiple genes or by the interaction between genes, regions, and ethnic groups. Certain limitations of this study were as follows: (1) the subjects of the study were limited to the population of Guangxi in China, and the sample size was small. Thus, studies with varied ethnic groups and regions and large sample size are needed. (2) Selective bias was possible, and significant differences in clinical data between the IS and control groups were found. In addition, potential risk factors for stroke existed in people in the control group. (3) The pathological mechanism underlying the genetic polymorphism of *miR-499A>G* and ischemic stroke was still unclear. All genetic mutations in *miR-499* and other factors might cause stroke, which had an impact on the explanation of the results of this study.

In conclusion, this study suggested that the genetic polymorphism of *miR-499*A>G was associated with IS in the population of Southwest region of Guangxi in China, and G allele and AG genotype of *miR-499* (rs3746444) could increase the risk of IS. The molecular mechanism underlying the pathogenesis of IS and the genetic polymorphism of miR-499A>G in different ethnic groups need further exploration.
